# Immune Checkpoint Inhibitors for Patients With Preexisting Autoimmune Neurologic Disorders

**DOI:** 10.1001/jamanetworkopen.2025.13727

**Published:** 2025-06-04

**Authors:** Kylie Fletcher, Marc Machaalani, Razane El Hajj Chehade, Amin H. Nassar, Rashad Nawfal, Michael Manos, Alexander M. Menzies, Frank Aboubakar-Nana, Jessica C. Hassel, David J. Pinato, Alexandra Johnson, Anna C. Olsson-Brown, Matteo S. Carlino, Andrea Malgeri, Alessio Cortellini, Aditi Singh, Kaushal Parikh, So Yeon Kim, Abdul Rafeh Naqash, Georgina V. Long, Pavan Challa, Toni K. Choueiri, Elad Sharon, Shailee Shah, Douglas B. Johnson

**Affiliations:** 1 Vanderbilt University School of Medicine, Nashville, Tennessee; 2 Dana Farber Cancer Institute, Boston, Massachusetts; 3 Yale University, New Haven, Connecticut; 4 Melanoma Institute Australia, The University of Sydney, Sydney, New South Wales, Australia; 5 Royal North Shore Hospital, Sydney, New South Wales, Australia; 6 Mater Hospital, Sydney, New South Wales, Australia; 7 Service de Pneumologie, Cliniques Universitaires Saint-Luc, Brussels, Belgium; 8 University Hospital Heidelberg, Heidelberg, Germany; 9 Department of Surgery & Cancer, Imperial College London, Hammersmith Hospital, London, United Kingdom; 10 Division of Oncology, Department of Translational Medicine (DIMET), University of Piemonte Orientale, Novara, Italy; 11 Clatterbridge Cancer Centre NHS Foundation Trust, Liverpool, United Kingdom; 12 Westmead and Blacktown Hospitals, Sydney, New South Wales Australia; 13 Fondazione Policlinico Universitario Campus-Biomedico, Roma, Italy; 14 Department of Medicine and Surgery, Università Campus Bio-Medico di Roma, Roma, Italy; 15 Department of Oncology, Mayo Clinic, Rochester, Minnesota; 16 Medical Oncology, Stephenson Cancer Center, The University of Oklahoma Health Sciences, Oklahoma City; 17 Department of Neurology, Northwestern University, Chicago, Illinois; 18 Department of Neurology, Vanderbilt University Medical Center, Nashville, Tennessee; 19 Department of Hematology/Oncology, Vanderbilt University Medical Center, Nashville, Tennessee

## Abstract

**Importance:**

Immune checkpoint inhibitors (ICIs) are efficacious in many cancer types but can produce immune-related adverse events (irAEs). As such, patients with preexisting autoimmune disorders are often excluded from clinical trials, although subsequent studies have shown that many of these patients have acceptable ICI tolerance. The safety and efficacy of ICIs among patients with preexisting neurologic autoimmune disorders (NAIDs) is not well characterized.

**Objective:**

To evaluate the safety and clinical outcomes associated with ICI therapy among patients with NAIDs.

**Design, Setting, and Participants:**

This multicenter retrospective cohort study included patients with cancer who were treated with ICIs between October 2013 and May 2023 and had preexisting multiple sclerosis (MS), myasthenia gravis (MG), Guillain-Barré syndrome (GBS), and other NAIDs as well as a control cohort of patients with Parkinson disease (PD).

**Exposure:**

ICI therapy.

**Main Outcomes and Measures:**

Demographic and clinical characteristics (neurologic disability, active or recent immunosuppression), ICI outcomes (response, progression-free survival [PFS], and overall survival [OS]), and safety outcomes (NAID exacerbation, irAEs) were collected.

**Results:**

A total of 135 patients were included; the median (range) age was 72 (40-88) years, 84 (62%) were men, and 51 (38%) were women. A total of 45 patients had MS; 18, MG; 10, GBS; 5, another NAID; and 57, PD. Exacerbations occurred most frequently in MG (12 of 18 patients [67%]), often resulting in hospitalization (6 [50%]) or death (2 [17%]), with much lower rates in the MS cohort (8 of 45 patients [18%]). Ten patients with a history of GBS tolerated ICI without exacerbations, although 1 developed a fatal case of Lambert Eaton myasthenic syndrome following ICI treatment. No differences in response rate, PFS, or OS were observed between NAID groups.

**Conclusions and Relevance:**

In this cohort study of ICI use in NAIDs, patients with MG had frequent and more severe exacerbations, while those with MS had few exacerbations. No obvious differences in survival between groups were observed. ICI may be an option for many patients with appropriate oncologic indications and preexisting NAIDs.

## Key Points

**Question:**

Are immune checkpoint inhibitors (ICIs) safe for patients with preexisting neurologic autoimmune disorders (NAIDs), and what are the outcomes of ICI therapy in this patient population?

**Findings:**

In this cohort study with 135 participants, neurologic disorder exacerbations occurred most frequently in patients with myasthenia gravis, with modest rates of exacerbations in other diseases, including multiple sclerosis. No differences in response rate, progression-free survival, or overall survival were observed between NAID groups.

**Meaning:**

These findings suggest that ICIs may be an option for many patients with appropriate oncologic indications and preexisting autoimmune neurologic disorders, although they should be used with caution in patients with myasthenia gravis.

## Teaser

This cohort study evaluates the safety and clinical outcomes of immune checkpoint inhibitor therapy among patients with neurologic autoimmune disorders, including multiple sclerosis, myasthenia gravis, and Guillain-Barré syndrome.

## Introduction

Immune checkpoint inhibitors (ICIs) are efficacious across multiple cancer types. These agents block negative regulatory checkpoints restraining T cell activation that unleash cytotoxic cancer cell death, but they produce autoimmune-like syndromes that may impact any organ system.^1,2^ As such, patients with preexisting autoimmune disorders have been largely excluded from clinical trials testing ICI agents. However, several retrospective studies have shown that ICIs are generally tolerable in most patients with autoimmune diseases, with modest rates of autoimmune disease exacerbations and classical immune-related adverse events (irAEs).^3,4,5,6^ While paraneoplastic or de novo neurologic diseases are well-recognized complications of ICI therapy, patients with preexisting neurologic autoimmune disorders (NAIDs) remain a conundrum for clinicians. The effect of ICIs on baseline neurologic disease, and whether effects represent an exacerbation of known neurologic deficits or the development of new symptoms and inflammatory disease, is not well described.^7,8^

Exacerbations of an NAID can pose life-threatening risks in many disorders (eg, myasthenia gravis [MG]), with reports suggesting frequent flares in patients receiving ICIs.^9^ Furthermore, patients with NAIDs may be at higher risk of neurologic irAEs.^10^ However, NAIDs encompass a range of disorders that greatly vary in their underlying pathophysiology and clinical manifestations and which evolve over a patient’s lifetime, such as in multiple sclerosis (MS). These diseases can also range from monophasic (eg, Guillain-Barré syndrome [GBS]) without known long-term autoimmune risk to chronic and at risk of relapse (eg, MG); thus, better understanding the risk of ICI-related relapse in different diseases is necessary. Here, we aimed to better characterize the safety and clinical outcomes of ICIs in a multicenter study of oncology patients with comorbid NAIDs and other neurologic diseases (specifically Parkinson disease [PD]).

## Methods

### Ethical Considerations

This retrospective, multicenter cohort study was approved by the institutional review board at Vanderbilt University Medical Center and granted an exemption for informed consent due to use of only deidentified patient data. The study follows the Strengthening the Reporting of Observational Studies in Epidemiology (STROBE) reporting guidelines.

### Case Selection and Inclusion Criteria

All patients with an existing NAID were identified using institutional databases at 9 institutions. Patients were treated between January 1, 2015, and June 1, 2023. The cutoff date for follow-up was May 1, 2024. NAIDs included MS, MG, and GBS; transverse myelitis, nonparaneoplastic Lambert-Eaton myasthenic syndrome (LEMS), and multifocal motor neuropathy were categorized separately due to small sample size. A non–neuro-immunologic disease control group of patients with PD receiving ICI treatment was also identified. All patients received at least 1 dose of ICI therapy (specifically anti–programmed cell death-1 [PD-1] or anti–programmed cell death ligand 1 [PD-L1]–based therapy) and had evaluable follow-up for clinical and safety outcomes. Patients with preexisting paraneoplastic neurologic disease were excluded.

### Data Collection

Among eligible patients, demographic characteristics, cancer type, cancer outcomes (eg, response, progression, overall survival [OS]), NAID characteristics (prior neuro-immunological treatments, baseline disability, and duration of illness), and safety outcomes (flares of NAID, types and grade of irAEs including neurologic irAEs) were manually collected from the electronic medical records.

### Variables and Outcomes

Outcomes of interest were objective response rate, as measured by Response Evaluation Criteria in Solid Tumors (RECIST) version 1.1, as provided by the investigators; progression-free survival (PFS), defined as time from treatment start to progression by RECIST 1.1; and OS, defined as time from treatment start to death or last follow-up. Other outcomes included irAE incidence rate and severity as determined by Common Terminology Criteria for Adverse Event (CTCAE) version 5.0 and rate of exacerbation of preexisting NAID as determined by CTCAE.

To quantify the severity and baseline disability of the preexisting NAID, we used the following scales. The modified Rankin Scale (mRS) was used to measure degree of neurologic disability. Cognition was graded as either 1, impaired; or 2, not impaired. Ambulatory function was graded as 0, no gait assistance; 1, cane or unilateral assistance; 2, walker or bilateral assistance; 3, wheelchair; and 4, bedbound. Bladder impairment was graded as 0, none; 1, mild; 2, moderate (intermittent catheterization or manual compression to evacuate bladder); and 3, severe (almost constant catheterization). These metrics were usually not quantified in medical records and were extracted by investigators when possible.

### Statistical Analysis

We used descriptive statistics, medians, and ranges, to summarize treatment outcomes, NAID exacerbations, and irAEs. Kaplan-Meier survival curves were created to demonstrate PFS and OS and compared between groups using log rank testing. The response rate was expressed in terms of proportions and SDs; comparisons between disease cohorts was done using χ^2^ analysis. A 2-sided *P* < .05 were considered statistically significant. Prism version 10 was used to conduct analyses (GraphPad).

## Results

### Demographic Characteristics

We identified 135 patients; the median (range) age was 72 (40-88 years) years, 84 (62%) were men, and 51 (38%) were women. A total of 78 patients (58%) had an NAID (45 with MS; 18, MG; 10, GBS; and 5, other) and 57 (41%) patients had PD. Forty patients had melanoma, 19 non–small cell lung cancer (NSCLC), 16 urothelial or bladder cancer, 13 squamous cell carcinoma of various primary sites, 7 small cell lung cancer (SCLC), 7 renal cell carcinoma, and 33 other cancers. Twenty patients (15%) had stage I to III disease, and 115 (85%) had stage IV disease. Seventeen patients (13%) received combination ICI therapy and 118 (87%) monotherapy (Table 1).

**Table 1.** Population Demographic and Clinical Characteristics

Characteristic	Patients, No. (%)				
	MS (n = 45)	MG (n = 18)	GBS (n = 10)	Other (n = 5)^a^	Parkinson (n = 57)
Age, median (range), y	67 (46-83)	72.5 (62-88)	70.5 (51-85)	73 (52-84)	76 (58-88)
Sex					
Female	27 (60)	6 (33)	2 (20)	3 (60)	13 (23)
Male	18 (40)	12 (67)	8 (80)	2 (40)	44 (77)
Stage IV disease	41 (91)	16 (89)	9 (90)	5 (100)	44 (77)
Treated with ICI monotherapy	38 (84)	18 (94)	8 (80)	5 (100)	52 (91)
Disease duration, median (range), y	21 (2-52)	14 (2-40)	10 (1-35)	26 (6-45)	4 (0.1-16)
Time since last exacerbation, median (range), y	10 (0.4-27)	13.5 (0.1-40)	10 (1-35)	6 (1-36)	NA
Baseline mRS, median (range)	1 (0-5)	0 (0-3)	0 (0-4)	0 (0-4)	1 (0-4)
Previous hospitalizations	5 (11)	4 (22)	3 (38)^b^	0	2 (4)
Immunosuppressive treatment within last 3 mo	13 (29)	11 (61)	1 (10)	1 (20)	NA

Abbreviations: GBS, Guillain-Barré syndrome; ICI, immune checkpoint inhibitor; MG, myasthenia gravis; mRS, modified Rankin Scale; MS, multiple sclerosis; NA, not applicable.

^a^ Transverse myelitis (2 patients), autoimmune encephalitis, Lambert-Eaton myasthenic syndrome, and multifocal motor neuropathy (1 patient each).

^b^ Two patients’ previous hospitalizations were unknown.

### Baseline Disease Characteristics

Baseline neurologic disease characteristics were heterogeneous (Table 1). Overall, patients with PD and MS had the most functional impairment at baseline, whereas those with MG and GBS had less baseline disability. Wheelchair or bedbound status was documented in 3 of 33 patients with MS (9%), 0 of 15 patients with MG, 1 of 8 patients with GBS (13%), and 6 of 48 patients with PD (13%). Only 1 patient with an NAID had documented cognitive impairment (1 patient with MG had mild impairment of unclear relation to MG); 9 patients with PD (16%) had impairment. Overall, 25 patients from all NAID cohorts (32%) were receiving immunosuppressive or disease-modifying treatment at or within 3 months of ICI initiation; most often patients with MS (29%) or MG (61%). Among patients with MG with evaluable data, 5 of 10 (50%) had positive acetylcholinesterase antibodies.

### Safety: irAEs and Exacerbations

Neurologic irAEs due to the underlying NAID or PD (exacerbation of the underlying disease and development of new inflammatory disease), neurologic irAEs not due to the underlying neurologic condition, and nonneurologic irAEs and other safety outcomes are summarized in Table 2.

**Table 2.** Immune-Related Adverse Events and Disease Exacerbations

Adverse event	Patients, No. (%)					*P* value^a^
	MS (n = 45)	MG (n = 18)	GBS (n = 10)	Other (n = 5)	Parkinson (n = 57)	
Neurologic disease exacerbation	8 (18)	12 (67)	0	0	12 (21)	<.001
Exacerbation due to new inflammatory disease, No./total No. (%)	2/8 (25)	12/12 (100)	0	0	NA	<.001
Exacerbation due to recrudescence, No./total No. (%)	6/8 (75)	0/12 (0)	0	0	12/12 (100)	NA
Neurologic irAE other than NAID/PD	1 (2)	3 (17)	2 (20)	0	0	NA^b^
Hospitalized	2 (4)	6 (33)	2 (20)	0	2 (4)	NA
Required steroids for NAID	4 (9)	7 (39)	2 (20)	0	NA	.01
Non-neurologic irAE	16 (36)	8 (44)	7 (70)	4 (80)	25 (44)	.78
Deaths related to neuro event	1 (2)	2 (11)	1 (10)^c^	0	0	NA^b^

Abbreviations: GBS, Guillain-Barré syndrome; irAE, immune-related adverse event; MG, myasthenia gravis; MS, multiple sclerosis; NA, not applicable; NAID, neurologic autoimmune disorder.

^a^ Excluding other due to small numbers.

^b^ Analysis not performed due to insufficient sample size.

^c^ Death due to Lambert-Eaton Syndrome rather than GBS flare.

#### MS

Of 45 patients, 8 (18%) experienced new or recrudescence of symptoms related to their MS. Six of 8 (75%) were thought to be pseudo-exacerbations due to worsening of preexisting symptoms (weakness, diplopia, spasticity, gait instability). New symptoms included optic neuritis and altered mental status in 1 patient and hemiparesis in the other. For example, a patient in their 50s not receiving prior disease-modifying therapy presented with new vision loss and encephalopathy and had contrast-enhancing lesions in the optic nerves and more than 10 constrast-enhancing intraparenchymal lesions consistent with MS. Magnetic resonance imaging (MRI) was performed in 5 patients with neurologic changes, 3 of whom (60%) showed new or enhancing lesions; although, new lesions in a patient younger than 50 years (described later) did not correlate with clinical symptoms (worsened spasticity). Two patients had radiologically isolated syndrome (no history of symptomatic MS; only disease noted on imaging); neither developed neurologic symptoms while receiving ICI therapy or evaluable follow-up. Neurologic disease exacerbations after ICI were similar in patients receiving MS disease-modifying agents prior to and during ICI administration (2 of 13 [15%]) vs those not receiving these therapies (6 of 32 [19%]) (*P* = .79). One patient each had encephalitis and polymyalgia rheumatica not related to MS. Three of 8 patients (38%) with exacerbations of MS symptoms discontinued ICI treatment early. One patient was rechallenged and did not relapse. Two of 8 patients (25%) had severe symptoms (grade 3 and 4) and required hospitalization. Four of the 8 patients (50%) with neurologic irAEs did not return to baseline after treatment, including 1 patient with ongoing weakness who died due to an aspiration event 2 months after discontinuing nivolumab. Other, nonneurologic irAEs were observed in 16 patients (36%), most commonly colitis (4 patients) and hepatitis (3 patients).

Of note, only 3 patients in the MS cohort were younger than 50 years; only 1 of them had worsening MS symptoms, specifically spasticity and pain and a new demyelinating lesion on MRI without associated symptoms. This patient was able to continue receiving ICI treatment with intermittent low-dose steroids and increased pain medication to manage symptoms and was alive more than 5 years after treatment initiation.

#### MG

Among 18 patients, 12 (67%) experienced MG relapses; 6 of 12 (50%) were grade 3 or 4. Five of 8 patients (63%) receiving immunosuppression had exacerbations, whereas 6 of 8 patients (75%) receiving pyridostigmine only or no MG treatment had exacerbations (*P* = .59; 2 patients unknown). Three patients were tapered off their MG immunosuppressive treatment in the 3 months prior to ICI initiation and 2 (67%) relapsed. Defining features of exacerbations included dysphagia, dyspnea, diplopia, generalized muscle weakness, and dysarthria. Six of 12 patients with flare (50%) were hospitalized due to their flare (Table 3) and received acute immunosuppressive therapy; 1 patient had concurrent myocarditis/myositis and another a concurrent demyelinating inflammatory neuropathy. Ten of 18 patients (56%) discontinued ICI treatment due to MG exacerbation. One patient who had tapered off their azathioprine prior to ICI was rechallenged after restarting azathioprine, without an MG relapse and without oncologic progression at last follow-up. Two patients died from MG flares; 1 patient was a man in his 70s with SCLC treated with pembrolizumab with a 14-year history of MG and multiple prior treatments. He had an exacerbation of his MG and developed concomitant myositis and myocarditis 6 weeks into treatment, resulting in severe paraplegia and arrythmias. The other was a man in his 60s with colon cancer treated with pembrolizumab with a 4-year history of MG who developed progressive weakness and respiratory failure 2 weeks into treatment. An electromyography was performed on this patient and showed a demyelinating polyneuropathy, and the patient transitioned to comfort care. All patients who survived had complete recovery from their MG flare. One patient without MG exacerbation developed a separate neurologic irAE (inflammatory neuropathy). Other nonneurologic irAEs were recorded in 8 of 18 patients (44%), notably myocarditis (2 patients, including 1 additional grade 4 event with full recovery), grade 3 pericarditis, and colitis or enteritis (2 patients).

**Table 3.** Survival Outcomes

Outcome	MS	MG	GBS	Other	Parkinson
Median PFS, mo	9.6	15.8	10.6	Not reached	9.1
Median OS, mo	20.1	33.5	Not reached	Not reached	18.7
Response rate, No./total No. (%) [SD, No.]	16/41 (39) [11]	3/14 (21) [5]	2/10 (20) [2]	4/5 (80) [1]	22/49 (45) [8]

Abbreviations: GBS, Guillain-Barré syndrome; MG, myasthenia gravis; MS, multiple sclerosis; OS, overall survival; PFS, progression-free survival.

#### GBS

Of these 10 patients, diagnosis of GBS occurred a median (range) of 10 (1-35) years prior, and 3 patients (33%) had residual neurologic deficits. Two patients with GBS had neurologic complications; a man in his 50s with NSCLC, who had GBS 9 years prior, developed worsening weakness in the setting of polymyalgia rheumatica and was hospitalized and treated with steroids with rapid recovery. A woman in her 80s with NSCLC and severe GBS 11 years prior developed new lower extremity weakness and was diagnosed with paraneoplastic LEMS 2 months after starting pembrolizumab. This patient was hospitalized and died due to neurologic complications. No other patients experienced flares or other neurologic irAEs. Seven patients (70%) had other nonneurologic irAEs, including hepatitis (1 patient) and rash (2 patients).

#### Other NAIDs

A total of 5 patients had other NAIDs, including nonparaneoplastic LEMS, 2 patients with isolated transverse myelitis, and autoimmune encephalitis (occurred 26 years prior). No patients in this group developed neurologic irAEs, although 4 (80%) had nonneurologic irAEs including enteritis or colitis (2 patients) and hematologic abnormalities (1 patient).

#### PD

Of 57 patients, 12 (21%) appeared to have worsening of existing parkinsonian symptoms after starting ICI. Two patients (4%) were hospitalized, and only 1 patient discontinued ICI due to worsening gait impairment resulting in a hip fracture. Other irAEs were observed in 22 patients (39%), most notably colitis (9 patients), hepatitis (6 patients), and myocarditis (2 patients, including 1 fatal case). No other neurologic irAEs were noted. No patients died directly from complications of their PD.

### Clinical Outcomes

There was no difference in PFS or OS between groups (Table 3 and Figure). Patients with PD, who were older and with higher baseline disability, had the numerically lowest PFS and OS. The response rate did not statistically differ between groups . When specifically comparing patients with an NAID with those with PD, PFS (median, 12.9 months vs 9.1 months; hazard ratio [HR], 0.87; 95% CI, 0.56-1.34; *P* = .65), OS (median, 33.5 months vs 18.7 months; HR, 0.81; 95% CI, 0.50-1.32; *P* = .40), and nonneurologic irAE rate (32 of 74 [43%] vs 25 of 57 [44%]; *P* = .94) were not statistically different between groups.

**Figure.**
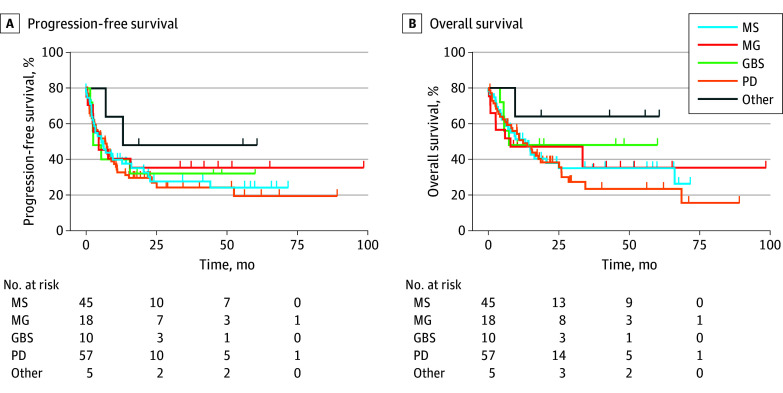
Survival Outcomes (A) Progression-free survival and (B) overall survival in patients with neurologic autoimmune disorders myasthenia gravis (MG), multiple sclerosis (MS), Guillain-Barré syndrome (GBS), Parkinson disease (PD), and other neurologic autoimmune disorders.

## Discussion

To our knowledge, this study is the largest to characterize the safety and clinical outcomes of ICIs in patients with an NAID. We observed that exacerbations of NAID in patients receiving ICIs varied significantly between different underlying disease states. Patients with MG had high rates of exacerbations (often clinically severe) with less frequent exacerbations in other disease cohorts. Additionally, clinical outcomes associated with ICI therapy did not vary by preexisting NAID, and durable responses were observed in some patients.

Two-thirds of patients with MG had exacerbations after receiving ICI therapy. Half required hospitalization, 2 of 18 (11%) died of their neurologic disease, and another patient had a severe exacerbation and fulminant cancer progression, both of which contributed to his death. However, the patients who responded to ICI treatment did so durably, and several patients had long-term stable disease, resulting in approximately 40% of patients alive and progression free beyond 2 years. These data are consistent with published case reports showing high risk of MG exacerbations but promising antitumor efficacy.^9^ Thus, the substantial risk of toxic effects should be carefully balanced with potential for positive outcomes, particularly in patients without other treatment options and potentially responsive tumors.

Exacerbations of other NAIDs were uncommon and consistent with prior data, which is likely due to the significant heterogeneity in underlying disease pathophysiology in patients with an NAID.^4,5,9,11,12,13,14^ Thus, risk-benefit discussions should be tailored to the specific underlying neurologic disease and should involve multidisciplinary, disease-specific discussions between patients and their neurology and oncology clinicians.

No definitive recurrence of GBS was noted in the 10 patients with a history of GBS, although none had recent or active GBS. This is in line with prior data suggesting that patients with historic GBS are not at greater risk of further exacerbations over other populations.^15,16^ One patient with a history of GBS developed paraneoplastic LEMS, and another patient developed polymyalgia rheumatica and was treated for her worsening weakness. This may suggest an underlying predisposition to autoimmunity overall.^17,18^

MS relapses rates were also low and consistent with prior results.^19^ One patient with severe MS at baseline aspirated and had respiratory failure after discontinuing anti–PD-1 treatment; this was thought to be at least partially related to an MS pseudo-exacerbation. Patients with clinically or radiographically inactive MS, typically older patients who no longer required immunosuppressive disease-modifying therapy, may have recrudescence of prior symptoms in the presence of systemic illness or injury as a result of the prior neuronal damage.^20^ These patients did not present with new clinical relapses, as evidenced by new radiographic or clinical activity not previously identified and represented the majority of the patients with disease exacerbation in this cohort. In 3 patients younger than 50 years, whose disease was more active and inflammatory in nature, only 1 patient experienced worsening of the prior neurologic deficits without radiographic changes and continued ICI with a durable response. Our data reassure us that patients with clinically inactive MS in later stages of their disease, especially those not requiring ongoing immunosuppressive therapy, have a low risk of recurrent inflammatory neurologic disease activity.

Of note, a small subset of patients with PD who received ICIs experienced worsening parkinsonian symptoms. Given that patients with PD frequently have disease exacerbations in the context of systemic illnesses,^21^ we suspect that PD worsening was due to systemic disease and not a direct consequence of the ICI-related T cell activation. It is possible that dosage and absorption of PD medications were affected in the subset of patients who developed comorbid nonneurologic irAEs such as colitis. Importantly, counselling and neurology consultation should be considered in patients with PD to optimize functional status prior to ICI initiation.

Identifying which patients with neurologic disease are likely to experience exacerbations with ICI use remains a major challenge in clinical practice. In our cohort, neurologic disease exacerbations were specific to the underlying disease. Patients with monophasic conditions (such as GBS), those with MS (who were older with less inflammatory disease), and those with more predictably noninflammatory disease, such as PD, had low risks of exacerbation. Patients with MG, a disorder that often requires some degree of immunosuppressive therapy throughout the disease course, had the highest risk of disease exacerbation or mortality. However, other factors may have contributed^22^; for example, patients with MG exacerbations had worse baseline neurologic disability at ICI initiation. Relapse rates were slightly higher (although not statistically significant) in patients with MG and MS who were not receiving immunosuppressive therapies as well; it is possible that optimization of the underlying neurologic disease may minimize neurologic irAEs. Patients at higher risk may also have different populations of quiescent T cells or circulation autoantibodies present that are unleashed by ICI therapy. Long-term detection using disease biomarkers before and after ICI may help better identify patients most at risk.^23^ Further stratification by individual disability levels, disease characteristics, and treatment type may help better understand which patients are at the highest risk and who can be safely treated with close monitoring.

### Limitations

This study has limitations, including its retrospective nature and small number of individual neurologic disease cohorts. Additionally, neurologic disease exacerbations often co-occurred with other irAEs and with cancer progression, making symptom attribution challenging in some cases, particularly when center-specific diagnostic testing and management strategies varied. Another limitation of this study is the paucity of laboratory, electrodiagnostic, and radiographic biomarker data available due to the multicenter design, with varying degrees of neurology expertise available at each center. Additionally, it is possible that patients with severe or aggressive preexisting NAIDs were not captured in this cohort due to concerns for neurologic disease exacerbation resulting in forgoing ICI.

## Conclusions

In this cohort study of patients with NAIDs who were treated with ICI therapy, rates of disease exacerbation were low in patients with preexisting autoimmune neurologic diseases aside from MG. Nevertheless, close monitoring of patients with an NAID, particularly those with known active disease, should be considered when receiving ICI. Comanagement between oncology and neurology is needed prior to ICI initiation for risk stratification and baseline disease assessment and optimization. Ongoing multidisciplinary care may help minimize the effects of potential disease exacerbations. ICI therapy can be considered in cases of preexisting NAID on a case-by-case basis and requires a careful risk benefit discussion.

## Notes

Supplement.
Data Sharing Statement
