# Supplementary material for: Immune Checkpoint Inhibitors for Patients With Preexisting Autoimmune Neurologic Disorders
Source: JAMA Netw Open. 2025 Jun 4;8(6):e2513727. doi: 10.1001/jamanetworkopen.2025.13727 (PMC12138719; doi:10.1001/jamanetworkopen.2025.13727)
Supplement: Supplement. — Data Sharing Statement [file jamanetwopen-e2513727-s001.pdf]

Data Sharing Statement

Fletcher. Immune Checkpoint Inhibitors and Patients With Preexisting Autoimmune Neurologic Disorders. *JAMA Netw Open*. Published June 04, 2025.
doi:10.1001/jamanetworkopen.2025.13727

Data
Data available: Yes
Data types: Deidentified participant data
How to access data: douglas.b.johnson@vumc.org
When available: With publication

Supporting Documents
Document types: None

Additional Information
Who can access the data: researchers whose proposed use of the data has been approved
Types of analyses: Research purposes
Mechanisms of data availability: with signed DUA
